# Impacts of Different Processes on the Nutritional and Antinutritional Contents of White and Blue Lupin Seeds and Usage Possibilities for Sustainable Poultry Production

**DOI:** 10.3390/ani13223496

**Published:** 2023-11-13

**Authors:** Tugce Uzun, Aylin Agma Okur

**Affiliations:** Department of Animal Science, Agricultural Faculty, Tekirdag Namık Kemal University, Tekirdağ 59030, Turkey; tugce--uzun@hotmail.com

**Keywords:** lupin, quinolizidine alkaloids, germination, autoclaving

## Abstract

**Simple Summary:**

The search for alternative raw feed materials that can contribute to the solution of many different problems—such as meeting the food needs of people, soil quality, ecological sustainability, increasing profitability in animal production, positively affecting the quality of animal products, and revealing the nutritional content of these alternative raw materials—is an important challenge. Revealing the possibilities of using these alternative raw materials in animal nutrition is becoming increasingly important for researchers, producers, consumers, and our planet. Lupin seeds might be perceived from these perspectives as a quality raw plant material that contains high amounts of protein, fat, fiber, phenolic compounds, phytosterol, beta-carotene, antioxidant, tocopherol, oleic acid, linoleic acid, carbohydrate, oligosaccharide, calcium, iron, phosphorus, and vitamin E. The possible downsides of lupin seeds might be quinolizidine alkaloid, raffinose family oligosaccharide (such as raffinose, stachyose, and verbascose), and non-starch polysaccharide contents, which act as antinutritional factors when consumed by poultry. In this study, germination and autoclaving processes were applied. Their effects were investigated in order to reduce the quinolizidine group’s alkaloids in lupin grains. We suggest that germination might enhance the usage ratios of lupin seeds in poultry diets and may lead to an increase in the profitability of poultry enterprises.

**Abstract:**

In the current era, it is important to consider economic and ecological sustainability issues while optimally meeting the nutrient needs of poultry. The use and research of alternative feedstuffs have gained importance due to these factors. The aim of this study is to reveal the raw lupin seeds’ nutrient ingredients as an alternative feedstuff and the effects of debittering methods. In the present study, two different treatments (germination for 2 days; heat treatment in an autoclave at 130 °C for 20 min) were applied to white and blue lupin seeds, and the differences in nutrient compositions between them and raw seeds were determined. When fatty acid compositions were analyzed, oleic, γ-linolenic, arachidic, behenic, erucic, and lignoceric acid values were found to be the highest in the raw, autoclaved, and germinated forms of white lupin (*p* < 0.01). The highest values of palmitic, stearic, and linoleic acids were observed in blue lupin (*p* < 0.01). While the value of total quinolizidine alkaloids (QA) in raw white lupin grains was higher than 1.943 mg/g, it was higher than 1.800 mg/g in autoclaved and germination-treated grains. Similarly, the total QA value of raw blue lupin grains was 0.894 mg/g, 0.609 ± 0.244 mg/g in germination-treated seeds, and 0.705 ± 0.282 mg/g in autoclave-treated seeds. As a result of these findings, it can be said that the methods applied for the removal of bitterness gave promising results. Furthermore, it would be rewarding to use these lupin varieties in in vitro and in vivo experiments to reveal the impacts and mechanisms of debittering methods on poultry.

## 1. Introduction

In the poultry industry, one of the highest expenditure items is feed prices, which account for approximately 60–70% of operating costs [1,2]. Among feed raw materials, animal and plant protein sources constitute the nutrient sources that should be included in diets in order to meet the nutrient requirements of poultry, but their high prices attract attention [3]. Due to the high prices of animal protein raw materials and the ban on the use of chicken meal in poultry diets in the European Union, all countries, especially European countries, have turned to the use of plant protein sources in the preparation of poultry diets [4,5]. In addition, issues such as sustainability, environmental awareness, carbon footprint, and consumer demands are also influential in the preferences for the use of plant protein sources as feedstuffs. In addition, worldwide events, such as pandemics and wars, have been observed to negatively affect the poultry and feed sector in terms of price and production due to the difficulties in the procurement and transportation processes of raw feed materials. In addition to these reasons, the number of poultry birds is increasing, and the demand and need for raw materials rich in protein content are also increasing. There is also an increase in the consumption of raw materials, such as soy as human food, in proportion to the increase in population. The optimum utilization principle of the limited resources on our planet is also gaining importance in terms of ecology and sustainability. In order to meet demands arising from the consideration of all these needs and priorities, poultry producers and feed producers, especially scientists, have turned to the search for alternative feedstuffs that are rich in protein content [6,7,8,9,10,11].

In the search for economically and ecologically sustainable solutions, lupin is considered an important alternative, and it is estimated that its use in animal nutrition will become widespread in the future [4]. Studies have shown that lupin species have superior compositions and lower alkaloid amounts (below 0.001%) compared to previously produced results of species-breeding studies [12]. However, the use of sweet lupin varieties does not mean that the obtained grains would necessarily be from sweet varieties. There are bitter and/or wild lupin varieties in many parts of the world, and this might cause cross-pollination between these varieties and the sweet ones [13]. In addition, scientific studies have shown that these varieties can grow in environmental and soil conditions that other legume varieties cannot adapt to, and they provide soil improvement by fixing 18–35 kg/da nitrogen to the soil [14,15].

Lupin, which belongs to the Leguminosae family, is a plant that grows in the Mediterranean basin, and there are mostly white (*Lupinus albus*), blue (*Lupinus angustifolius*), and yellow lupin (*Lupinus luteus*) species in Turkey [4,15]. In general, lupin contains 28–45% of protein, 5–20% of fat, 30–40% of fiber, and 0–5% of starch, and it is rich in phenolic compounds, phytosterols, vitamin E, beta-carotene, tocopherol, calcium, iron, phosphorus, oleic acid, linoleic acid, carbohydrates, and oligosaccharides [12,16]. In addition, its antioxidant, gluten-free, superior water binding, and emulsification properties have made it an attractive raw feed material for poultry feed producers [12,15,16,17]. The only factor limiting its use in poultry feeding is the quinolizidine group’s alkaloids and glycosides it contains. These alkaloids (lupinine, lupanine, sparteine, 13α-hydroxylupanine, α-isolupanine, and angustifoline) in lupin create an antinutritional factor effect when consumed raw by animals [12,18,19]. These undesirable antinutritional factors can be removed or reduced by applying different processing conditions to lupin (autoclaving, extrusion cooking, boiling, scalding, boiling in water, frying, roasting, baking, pasteurization, sterilization, pressure cooking, microwave cooking, germination, soaking, fermentation, peeling, grinding, crushing, soaking, drying, freeze-drying, fermentation, the addition of various chemicals and enzymes, extraction by ultrasound, etc.) [3,16,20,21].

With respect to the soaking method, one of the traditional debittering methods, lupin seeds remain in high amounts of water for long periods, such as 2–4 days [21]. Comparingly high water use might not be sustainable. Additionally, boiling in water and roasting are the other common methods. However, the temperatures are high for long periods of time when using these methods. Temperatures higher than 120 °C with low humidity and for long durations might cause Maillard reactions besides nutrient loss [1,22]. In this study, we aim to determine changes in the nutrient contents of white and blue lupin varieties that are grown in the Tekirdağ region after heat and steam treatment (for 20 min at 130 °C and under 0.2 MPa of steam pressure in the autoclave) and germination (for 48 h in the dark). In this manner, we aim to reveal the possibilities of using lupin, which is a plant protein and oil source, as an alternative feedstuff that might help reduce feed costs in poultry production farms and the feed industry. 

## 2. Materials and Methods

### 2.1. Material

The white lupin (*Lupinus albus*) and blue lupin (*Lupinus angustifolius*) species used in the study were harvested in July 2021 (in Tekirdağ province of Turkey). 

### 2.2. Preliminary Experiment for Autoclave Process Times

A preliminary experiment was carried out to determine the duration of the autoclaving process. The seeds were autoclaved at a vapor pressure of 0.2 MPa and a temperature of 130 °C for 10 and 20 min. The results of the laboratory analysis of the obtained seeds were statistically compared, and the results of the grains subjected to the process in the autoclave for 20 min were statistically different (*p* < 0.01).

There was a statistically important reduction in the amounts of ether extracts (EEs), total sugar, total alkaloids, acid solvent insoluble lignin (ADL), 2,2-diphenyl-1-picrylhydrazl (DPPH), and L* (lightness) (*p* < 0.01) when increasing processing times in white lupin seeds. However, an increase was observed in metabolizable energy (ME), acid solvent insoluble fiber (ADF), 2,2′-Azino-bis-3-ethylbenzthiazoline-6-sulphonic acid (ABTS), phenolic compounds, a* (red-green), and b* (yellow-blue) levels (*p* < 0.01). 

Doubling the autoclaving time in blue lupin seeds increased crude protein (CP), crude cellulose (CC), ADF, a* (*p* < 0.01), and DPPH (*p* < 0.05) levels. Meanwhile, increasing processing times led to a decrease in starch, total sugar, total alkaloid, neutral solvent insoluble fiber (NDF), ADL, ABTS, phenolic compounds, L*, and b* levels (*p* < 0.01).

In addition to these results, the total quinolizidine levels were numerically decreased by increasing autoclaving times in both lupin species.

In the preliminary experiment, higher processing times were not preferred in order to reduce nutrient loss in grains and time and energy usage loss for producers.

### 2.3. Processes

The experiment was planned in order to determine the changes in nutrient contents and the antinutritional factors of raw, autoclaved (20 min), and germinated (48 h in the dark) white and blue lupin grains. The effects of these changes on the utilization of grains in poultry diets were investigated. 

The autoclaving process applied to white and blue lupin grains was carried out at a steam pressure of 0.2 MPa and a temperature of 130 °C for 20 min. The germination process applied to white and blue lupin grains was carried out by adding 3 times the amount of water to the grains, leaving them for 24 h, and then draining and leaving them to germinate in the dark for 48 h. In order to finalize germination, the grains were dried in an oven at 50 °C. The grains of all treatments were ground, sieved through a sieve with a sieve diameter of 1 mm, and stored in Ziplock polyethylene bags at +4 °C until analyses. Raw, autoclaved, and germinated white and blue lupin grains were analyzed for nutrients in triplicate, and the effects of the methods on the nutrient contents of lupin were determined.

### 2.4. Laboratory Methods

Dry matter (DM), crude ash (CA), crude protein (CP), ether extract (EE), and crude cellulose (CC) analyses of white and blue lupin samples were determined according to [23]. Neutral solvent insoluble fiber (NDF), acid solvent insoluble fiber (ADF), and acid solvent insoluble lignin (ADL) were determined according to Van Soest et al. [24]. The values were used to calculate cellulose and hemicellulose contents. The formulas used in the calculation are as follows [24] (Equations (1) and (2)).

Cellulose (g/kg dry matter) = ADF − ADL (1)

Hemicellulose (g/kg dry matter) = NDF − ADF (2)

A total sugar analysis was performed using the Luff–Schoorl method according to Akyıldız [25] and Cemeroğlu [26]. Starch analyses were determined according to Akyıldız [25]. Metabolizable energy (ME) values were calculated according to the equation of Carpenter and Clegg [27] (Equation (3)).

ME (kcal/kg) = [53 + 38(%CP + 2.25%EE + 1.1%Starch + %Sugar)](3)

The fatty acid profile was determined via gas chromatography (GC) (SHIMADZU GC-2010 Plus C120954, Kyoto, Japan) by preparing the methyl esters of the oil according to Garces and Mancha [28]. The GC instrument was operated in conjunction with a flame ionization detector (FID). The separation of 11 fatty acid standard mixtures was achieved on a TR-CN 100 (100 m × 0.2 mm × 0.25 mm) capillary column (Teknokroma, Sant Cugat del Vallès, Spain). The inlet temperature was set at 250 °C, and electron ionization (EI) was used. Helium was used as carrier gas with a flow of 30 mL/min (constant flow mode). The oven temperature program for the TR-CN 100 arm started at 100 °C, was rapidly increased to 240 °C at 3 °C increments per minute, and held at 240 °C for 10 min for a total of 60 min. The data were expressed as a percentage of the product mass [29,30]. 

A beta-carotene analysis was determined according to Chuah et al. [31] by reading the absorbance (Abs) value via a spectrophotometer (SHIMADZU UV-1208 A1012, Kyoto, Japan) that was adjusted to a wavelength of 449 nm. 

Phenolic compounds were analyzed according to the Folin–Ciocalteu method adapted from Singleton et al. [32] by reading the absorbance (Abs) on a spectrophotometer set at a wavelength of 765 nm. Gallic acid was used as a reference, and the results were expressed as mg gallic acid equivalent (GAE) per kg sample (mg GAE/kg sample). Calculations were carried out with the help of the gallic acid standard curve [21,26].

Two methods were used for antioxidant activity analysis. In the first method, the antioxidant levels were determined according to the DPPH method adapted from Dorman et al. [33] by reading the absorbance (Abs) value in a spectrophotometer adjusted to a wavelength of 517 nm. The results were calculated as radical scavenging activity (RSA) [21]. In the second method, the absorbance (ABS) value was determined according to the ABTS method adapted from Miller et al. [34] via a spectrophotometer adjusted to a wavelength of 734 nm [35].

Viscosity contents were determined via a cone plate Brookfield viscometer (LABGENI RV-1D, Changsha, China) according to the method adapted from Kaczmarek et al. [36,37], Şamlı et al. [38], and Konieczka [39]. 

Color analysis—L* (brightness), a* (red-green), and b* (yellow-blue) scale—values were measured via KONICA MINOLTA (CR-5 2101411, Tokyo, Japan) according to the CIE Lab color parameter scale [40]. 

A total alkaloid analysis was determined via titration according to Von Baer et al. [41]. The quinolizidine alkaloids (QA) analysis of samples was measured via LC-MS/MS by Eurofins WEJ Contaminants GmbH (Hamburg, Germany), an accredited laboratory for this analysis. Thus, the amounts of lupanine, 13α-hydroxylupanine, cytisine, sparteine, angustifoline, lupinine, multiflorine, and α-isolupanine quinolizidine alkaloids were determined. 

### 2.5. Statistical Design

The study was planned according to a 2 × 3 factorial design with two lupin varieties (white and blue lupin) and three treatments (control, germinated, and autoclaved seeds) using three replicates. The data obtained were subjected to Duncan’s multiple comparison test. Statistical analyses of the obtained data were performed using the SPSS program package [42]. The statistical modeling of the study is provided below:

Y_ijk_ = μ + S_i_ + O_j_ + (SO)_ij_ + e_ijk_ (4)

Y_ijk_: Observation value according to ith variety and jth treatment;

μ: Population mean;

S_i_: Effect of variety i;

O_j_: jth effect of treatment;

(SO)_ij_: Variety × effect of treatment;

e_ijk_: Error.

## 3. Results

Different debittering methods, such as soaking, dehulling, autoclaving, fermentation, cold plasma, ultrasound, and germination, were preferred and studied from past to present research in order to reduce the antinutritional factors of legumes. In this study, we aimed to choose a basic method that was easy to apply in a comparatively short period of time. Protein denaturation might be an important adverse effect of thermal methods, and heat source and duration should be carefully considered when applying these methods [43]. Because of these reasons, the germination method was chosen for comparison with autoclaving as a thermal treatment method.

### Nutrient Ingredient Changes in White and Blue Lupin Seeds via Processing

When the effect of cultivars on CP (DM %) levels was individually analyzed, the highest CP value was observed in white lupin (35.45%). In addition, when the effect of treatment alone was statistically analyzed, the highest CP (34.17%) value was observed in lupin seeds that were subjected to germination treatment (*p* < 0.01; Table 1).

The effects of lupin variety, treatment, and their interactions on CP (DM %) levels were found to be statistically significant (*p* < 0.01; Table 1). When the results were analyzed, it was observed that CP levels were the highest in germinated white and blue lupin grains (white lupin, 35.99%; blue lupin, 32.34%).

When the effect of cultivars on EE (% DM) levels was individually analyzed, the highest EE value was found in white lupin (8.316% DM). In addition, when the effect of treatments alone was statistically analyzed, it was observed that the EE (8.315% DM) value was the highest in lupin seeds subjected to autoclaving treatment (*p* < 0.01; Table 1).

The effects of lupin variety, treatment, and their interactions on EE (% DM) levels were found to be statistically significant (*p* < 0.01; Table 1). As shown in Table 1, the highest EE levels (white lupin, 9.911% DM; blue lupin, 6.719% DM) were found in autoclaved white and blue lupin grains.

In this study, the starch contents of raw white and blue lupin grains were found to be 4.781 and 6.987%, respectively. As shown in Table 1, the effects of lupin variety, treatment, and their interactions on total starch (%) levels were found to be statistically significant (*p* < 0.05). In terms of starch levels, no difference was observed between the raw and germinated forms of the same type of lupin samples, while the lowest numerical values were found in the autoclaved forms of both white and blue lupin grains (white lupin, 4.045%; blue lupin, 4.413%).

Table 2 shows the plant cell wall components of lupin grains, such as NDF (neutral detergent fiber), ADF (acid detergent fiber), and ADL (acid detergent lignin), in order to define the dietary fiber content. The NDF analysis’s results show hemicellulose, cellulose, lignin, and insoluble ash content. However, ADL only indicates the lignin content of the plant material. Additionally, hemicellulose, cellulose, and lignin contents can be found separately from the analyzed results:

Lignin (g/kg DM) = ADL;

Cellulose (g/kg DM) = ADF − ADL;

Hemicellulose (g/kg DM) = NDF − ADF [44,45].

**Figure 1:**
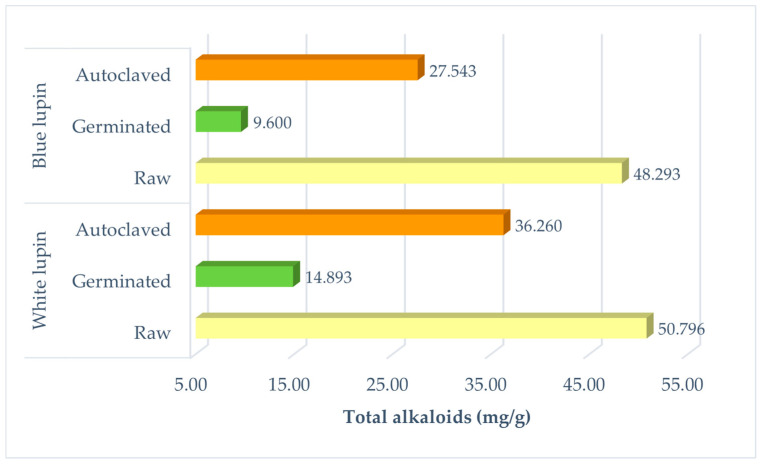
Total alkaloid (mg/g) levels of blue and white lupin seeds before and after debittering methods.

The total alkaloid levels ranged between 9.6 and 50.8 mg/g. While the total raw seed alkaloid levels were found to be the highest, the germinated ones were the lowest. The total alkaloid levels in white lupin seeds were reduced by approximately 70.7% after the germination process (Figure 1; *p* < 0.001). Also, similar effects were observed in blue lupin seeds, and the total alkaloid levels were decreased by 80.1% in germinated materials compared to the raw seeds (*p* < 0.001). Lupin varieties containing ≥10 mg/g DM alkaloid in seeds were identified as “bitter lupin” [46,47]. Keuth et al. [48] reported the total quinolizidine alkaloid levels as 20–21 mg/g with respect to two bitter lupin seeds varieties; simultaneously, 0.152 mg/g was reported for alkaloid levels relative to a sweet lupin variety. The QA analysis showed that the total alkaloid levels of untreated white and blue lupin seeds were found to be >1.943 mg/g and 0.894 mg/g, respectively (Table 3 and Table 4). 

After the application of debittering processes (germination and autoclaving), a numerical decrease was observed in both lupin species’ (white and blue lupin) seeds. Statistical analysis could not be performed due to the low number of replicates. However, it should be noted that the quinolizidine alkaloid content of raw blue lupin is lower than that of white lupin seeds. This is one of the issues that might have an impact on the level of use in poultry diets and should be taken into consideration.

The aim of this study was to determine both the nutritional and antinutritional characteristics of different lupin grain species as alternative and sustainable feedstuff. To meet these perspectives, the antioxidant capabilities of lupin seeds were examined via free radical scavenging activity in the context of DPPH (2,2-diphenyl-1-picrylhydrazl) and ABTS (2,2′-Azino-bis-3-ethylbenzthiazoline-6-sulphonic acid) levels (Table 5). DPPH is a stable nitrogen-centered free radical compound and is widely used in the evaluation of peptides and phenolic compounds and food antioxidant capacity [49]. The concentration of the sample that reduces the DPPH radical scavenging activity by 50% (IC50) is a commonly used parameter for measuring antiradical activity, and a low IC50 is a sign of high scavenging activity. In this study, the lowest IC50 values were observed in autoclaved white lupin seeds, while the highest ABTS levels were observed in the same groups (*p* < 0.01).

Legumes contain various phenolic compounds, such as flavonoids and phenolic acids [50]. Phenolic compounds are also considered responsible for antioxidant effects. The number of phenolic compounds in grains is negatively related to protein contents. However, in this study, we could not observe a negative relation between the protein and phenolic compound levels of the lupin seeds, although nutrient ingredients in plant seeds (total polyphenols, RFO, NSP amounts, and their impacts) might be affected by the variety, climatic conditions, growing location, and germination process of plant materials, according to Mareček et al. [51]. 

Antioxidants, phenolic compounds, β-carotene, and color analysis results showed that varieties, debittering processes, and their interactions have a statistically important impact on the studied lupin seeds and their nutrient ingredients (Table 5).

**Figure 2:**
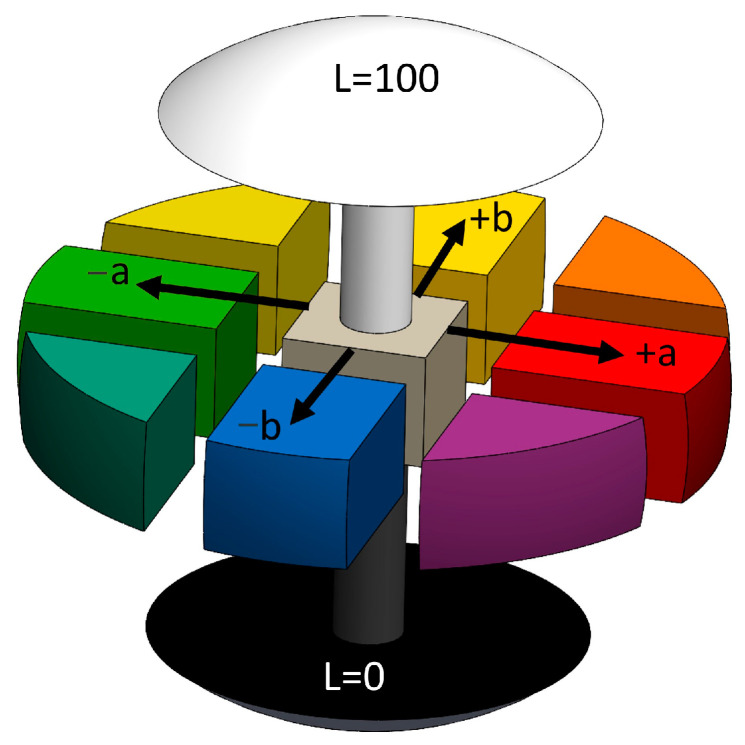
CIE Lab Color space in three dimensions.

The CIE Lab Color measurement system is a three-dimensional system that uses L* for lightness (levels from 0 to 100; a value of 100 means perfect black or absolute black), a* for redness/greenness (positive values represent redness), and b* for yellowness/blueness (positive values indicate yellow) (Figure 2). L*, a*, and b* values are the coordinates of a color point in the color space. 

**Figure 3:**
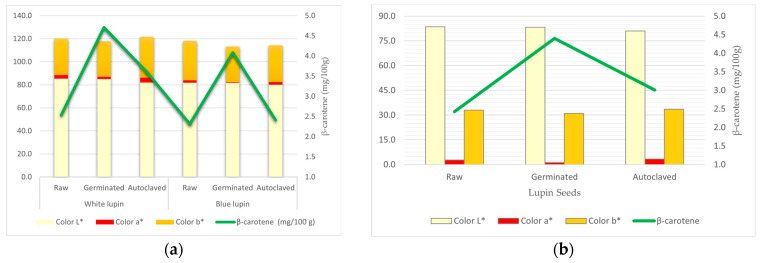
(**a**) Effects of interactions between the variety and process; (**b**) main effect of processes on lupin seeds, beta-carotene, and color measurements.

Germinated seeds tend to have higher amounts of DPPH, phenolic compounds, and beta-carotene. Simultaneously, lower redness (a*) and yellowness (b*) values were observed after the germination process (Table 5 and Figure 3, *p* < 0.01). The relation between the process, beta-carotene, and color changes is shown in Figure 3.

The fatty acid compositions of lupin seeds before and after the processes are shown in Table 6. According to the lupin species’ fatty acid content results, blue lupin seeds have the highest saturated (20.32%) and polyunsaturated (40.80%) fatty acid levels, while monounsaturated (38.88%) fatty acid levels were found to be the lowest. In untreated white lupin seeds, oleic, linoleic, and γ-linolenic acids are the most abundant fatty acids: 53.88%, 15.98%, and 12.51%, respectively. However, oleic, linoleic, and palmitic acids are indicated as the main fatty acids in raw blue lupin seeds: 38.88%, 37.99%, and 10.80%, respectively. The lupin varieties were statistically effective with respect to all fatty acid levels (*p* < 0.01). Additionally, Variety × Process interactions were found to be significantly effective as well.

## 4. Discussion

In this study, germination and autoclaving processes were applied, and their effects were investigated in order to remove and/or reduce the quinolizidine group’s alkaloids in lupin grains. Lupin seeds are identified as quality raw plant materials in terms of their nutrient content. However, quinolizidine alkaloids, which are specific to lupins, might also act as an antinutritional factor when poultry animals consume untreated seeds [43].

Alkaloids are secondary plant metabolites, and their main roles are to protect plants from extreme climate conditions (such as frost and drought), herbivores, bugs, and pathogens with toxic effects, bitterness, and umami taste. The presence of the umami taste could be linked with amino acids, especially glutamic acid, which is the amino acid with the highest amounts found in lupin varieties. Alkaloids that are specific to lupins are called “quinolizidine”. Quinolizidine alkaloids (QA) are generally biosynthesized from lysine amino acids (excluding some irregular QA). One of the di-amines, cad, is also a precursor of QA in the alkaloid biosynthesis pathway. Basic amino acids, biogenic polyamines, and QA comprise nitrogen reserves in plants [43,47,52,53].

The lupins categorized according to their QA contents are as follows:

<0.5 mg/g QA-containing lupins are identified as “sweet”;

0.5–1.0 mg/g QA-containing lupins are identified as “semi-sweet”;

1.0–2.0 mg/g QA-containing lupins are identified as “semi-bitter”;

>2.0 mg/g QA-containing lupins are identified as “bitter” [46,47,54,55].

Furthermore, Boschin et al. [55] stated that 0.5 mg/g of total quinolizidine alkaloids in white lupin seeds is decisive. In this context, even after debittering processes in this study, white lupins remained within the “semi-bitter” limits, and blue lupins remained within the “semi-sweet” limits.

In some studies, the maximum recommended use of lupin for broiler diets was reported to be 20% [56,57]. However, Kubis et al. [58] emphasized that the addition of up to 10% lupin to diets in poultry feeding can be safe and will not adversely affect performance. Additionally, Smulikowska et al. [59] indicated that lupin seeds should not be used in the starter diets of broilers. After the starter period, they also suggested using lupin seeds up to 15%.

In addition, other researchers have stated that white lupin can be added at up to 25% of the diet [60,61,62]. Some studies have even reported that the usage rate of white lupin in poultry diets can be increased up to 30–40% when the diet is supplemented with sulfur-containing amino acids such as methionine [63,64,65]. When determining the usage ratio of legumes as an alternative vegetable protein source in poultry diets, it will be beneficial to consider the types and amounts of tannins and alkaloids that are contained, in addition to the breed, age, and physiological state of the consuming animals [2]. Also, the toxicity of QA is affected by the lupin variety; environmental conditions; regions; soil *p* level; and climatic conditions, such as drought, rain, etc. [47,55].

The maximum value for the total QA of lupin in products used as human food is 0.2 mg/g. For animals, no clear information has been provided due to insufficient research, especially with respect to poultry, horses, and rabbits [66]. However, Boschin et al. [47] indicated that when the total quinolizidine alkaloid content of the diet was below 0.2 mg/g, no negative effects were observed in animals. In addition, the EFSA CONTAM Panel [67] reported the tolerable daily maximum QA levels for laying hens at 0.77–0.90 mg/kg live weight.

While the amount of CP in raw white lupin was 35.17%, it was observed to be 36.00% in white lupin treated with germination for two days and 35.20% in white lupin treated with steam pressure at 130 °C for 20 min in the autoclave. It was determined that the different treatments applied did not cause a statistically significant change in the amount of HP contained in white lupin grains. The CP content of raw blue lupin grains was 29.33%, whereas the CP content was 32.34% and 29.80% in two-day germination and autoclaved grains, respectively. This shows that the protein content of blue lupin grains after germination was statistically higher than the other treatments.

Erbaş et al. [68] reported that the CP content of white lupin was 32.2% in dry matter. Doxastakis et al. [69] reported that the CP content of white lupin was 32%. When other studies were examined, Tüzün [4] stated that the CP content (in dry matter) of lupin varied between 28 and 45%, and Yorgancılar et al. [12] stated that it varied between 33 and 47%. In his study, Çoban [70] stated that the CP content of white lupin in dry matter was 34%, and the CP content of blue lupin grains was 33.4%. Lampart-Szczapa et al. [71] revealed that the CP content of white lupin was 43.5%. In another study, it was determined that the CP contents of white lupin (Deşdiğin, Lolita, and Amigo genotype varieties) were 31.34%, 31.12%, and 30.06% in dry matter, respectively [15]. When all these studies are examined, it is observed that there is variation in terms of crude protein values. With respect to the main reason for this phenomenon, it should not be ignored that the protein levels contained in different lupin species and varieties may also differ. 

In another study, it was determined that the CP content of lupin was 41.3% in dry matter and decreased to 39.3% or ranged between 39.5% and 40.9% after the removal of bitterness [72]. In our study, unlike the study of Ertaş and Bilgiçli [20], no decrease in protein value was observed as a result of the bitterness removal process. Özcan [21] reported that the CP content of white lupin was 41.3% in his study, and it was reported that the CP content of white lupin decreased to 39.8% after the removal of bitterness with the classical extraction process; moreover, the CP content increased to 44.69% in dry matter after the removal of bitterness with the ultrasound extraction process.

Yorgancılar et al. [12] reported that the EE content of lupin varied between 5 and 20% DM. In addition, Tüzün [4] reported that the EE content of blue lupin varied between 4 and 7%, and the EE content of white lupin varied between 8 and 11%. Erbaş et al. [68] determined the EE content of lupin in dry matter to be 5.95% in their study. Similarly to Erbaş et al. [68], Kaya and Yalçın [18] reported that the EE content was 6% DM. The results of EE analyses in our study correspond with these studies.

In the study conducted by Doxastakis et al. [69], the EE content of white lupin was determined as 15.1% DM. Similarly, in the study conducted by Özcan [21], the EE content of white lupin was reported to be high (18.82% DM); the EE content decreased to 18.39% with the classical extraction (blanching) process, and the EE content was reported to be a minimum of 11.9% DM and a maximum of 22.01% DM using the ultrasound-assisted extraction process. The results obtained from our study differ from the results reported by Doxastakis et al. [69]. In addition, unlike the results reported by Özcan [21], the treatments applied to remove bitterness in this study did not cause a decrease in EE levels.

The starch content of lupin grains used In our study varied between 4.05 and 6.99%. However, Özcan [21] indicated that the starch content of white lupin was 10% and 12.6% in blue lupin, which is different from our results. Rahman et al. [72] reported higher starch contents at 16%. Unlike the results mentioned above, some studies determined that the starch content of lupin varied between 0 and 5% [12]. Similarly, Tüzün [4] stated that the starch content of lupin was 0.4% in his study. Our study results are similar to those reported by Yorgancılar et al. [12]. The differences observed between the starch contents of lupin seeds in previous studies [4,21,72] might be due to the differences in lupin seed species, varieties, debittering methods, and climate, soil, and storage conditions in which the product was grown and kept.

Crude fiber (CF) has generally been used as one of the main analysis methods for revealing the feed and feedstuff fiber content since the 19th century [44,73]. However, this method does not measure the soluble NSP. Because of this, neutral detergent (NDF) and acid detergent fiber (ADF) methods were developed and used as more detailed approaches. According to this point of view, ADL identifies “Lignin”. In addition, ADF reveals the cellulose and lignin contents of the analyzed feedstuffs. NDF reveals the attributes of the total “hemicellulose, cellulose, lignin” values of plant materials. However, dietary fibers comprise “Cellulose, Pectin, Hemicellulose, and Lignin”. With respect to a different definition, dietary fiber is the sum of the NSP and lignin contents of plant materials [44,45]. 

This study indicated that the blue lupin variety has the highest CF, NDF, ADF, and ADL levels compared to white lupin seeds. However, the germination process in white lupin seeds resulted in higher CF, NDF, and ADL contents compared to raw and autoclaved ones. While examining previous studies, it was observed that the CF content of lupin varied between 12 and 18% [4] and 13 and 14.4% [18] in dry matter. In addition to these, Guillon and Champ [74] indicated that the CF contents of lupins varied between 8 and 27.5% in dry matter. Additionally, Erbaş et al. [68] revealed that the CF content of white lupin was 16.2% in dry matter. Moreover, Tizazu and Emire [75] reported the CF content of lupin as 10.08%. The results of our study were similar to the aforementioned studies and ranged between 8.32 and 18.82. The ADF and NDF results of raw white and blue lupin seeds were observed to be similar to Jeroch et al. [76]. However, the ADL results of raw white lupin seeds were lower compared to those reported by Jeroch et al. [68].

Unlike our results, Özcan [21] reported that the CF content of lupin was 30%, van de Noort [77] reported it as 35–40%, and Yorgancılar et al. [12] stated that it was between 30 and 40%. It is thought that these differences between the crude fiber levels of lupin seeds may be attributed to the variety of seeds that were analyzed, the conditions in which they were grown (harvest year, climate, and soil and environmental differences), storage time and conditions, and debittering processes.

There were no differences between the treatments in terms of viscosity values in white lupin grains. In blue lupin grains, the highest viscosity (45.870 cP) was found in the germination treatment, and no differences were found between raw and autoclaving treatments.

When fatty acid compositions were analyzed, a significant difference was observed among lupin varieties. Oleic, γ-linolenic, arachidic, behenic, erucic, and lignoceric acid levels were higher in white lupin grains compared to blue lupin grains, and the difference was statistically significant (*p* < 0.01). However, palmitic, stearic, and linoleic acid levels were found to be higher in blue lupin grains (*p* < 0.01). Accordingly, total saturated and polyunsaturated fatty acid levels were higher, and monounsaturated fatty acid levels were lower in blue lupin grains. The fatty acid results of raw lupin seeds exhibited similarities with respect to Boschin’s [55] and Petterson’s [78] reports. 

It was observed that the effects of the lupin cultivar, applied methods, and their interactions significantly affected the results of antioxidant (ABTS and DPPH) and phenolic compound levels. DPPH is a stable free radical, and the lower the absorbance value, the higher the free radical scavenging activity of the antioxidant. When the two lupin varieties were compared in terms of phenolic compound contents, it was found that white lupin grains contained 1085.799 mg/L of phenolic compounds, and blue lupin grains contained 218.286 mg/L of phenolic compounds. In addition, the treatments (germination and autoclaving) caused a decrease in the phenolic compound content of blue lupin seeds. However, the phenolic compound contents of white lupin grains were not adversely affected by the autoclaving and germination processes. When the ABTS antioxidant analysis’s results were analyzed, it was similarly observed that the values of blue lupin grains were lower than those of white lupin grains. 

de Cortes Sánchez et al. [79] indicated that a high number of different physiological transformations can occur during the germination period. Due to these changes, the germination process might affect alkaloidal nitrogen mobilization. It might cause a reduction in the antinutritive substances (alkaloids, RFO, and phytic acids) of lupin seeds. Additionally, they suggested that germination should not exceed 3 days in order to prevent an increase in alkaloid levels and their transformation to alkaloid esters. 

## 5. Conclusions

Alkaloid levels are important and also cause restrictions when used in poultry diets. In this study, bitterness-reducing processes showed promising results with respect to lupin seeds, especially total alkaloid and QA levels. The germination of seeds for two days produced better results for all lupin varieties. This might lead to the use of lupin seeds in poultry diets as an alternative raw feed material. The reduction in and/or elimination of antinutritional factors can be achieved by applying different methods. According to the nutrient ingredients of lupin varieties, different results might be observed among the studies mentioned here. These differences might be related to lupin varieties, climate, geographic zones, season, and the land conditions of lupin plants. Because of these reasons, more studies are needed to reveal the effects of different debittering processes, and in vivo studies are also required in order to understand the impacts from all perspectives. However, in vivo studies can also encounter different results. The possible reasons for the variations in animal trials might be the experimental design, bird-rearing conditions, age, diet composition, nutrient ingredients, and processes of lupin seeds. Although the use of lupin cultivars in poultry diets has shown promising results, more studies on bitterness removal methods, including in vivo trials, are needed. However, it is thought that the use of lupin seeds—even as a small portion replacement of soybean meal, which is an imported product that is used as the main plant protein source in poultry diets—will have positive effects on economic and ecological sustainability; moreover, it can improve the quality of animal products by affecting unsaturated fatty acids and cholesterol in terms of their nutrient contents. The nutrient quality of poultry products (such as eggs, thighs, and breasts) should be the main topic for future studies on the use of lupin seeds because it affects consumers’ preferences and health.

## Figures and Tables

**Table 1:** Nutrient ingredients of white and blue lupin grains (part 1).

Main Effects	Treatments	DM (%)	Ash (%DM)	CP (%DM)	EE (%DM)	Starch (%)	Total Sugar (g/100 mL)	ME (kcal/kg)
White lupin	Raw	94.583 **c**	2.913 **ab**	35.168 **a**	6.997 **c**	4.781 **b**	0.653 **d**	2212.177 **b**
	Germinated	95.356 **a**	2.761 **ab**	35.998 **a**	8.041 **b**	4.781 **b**	0.845 **c**	2340.372 **a**
	Autoclaved	94.358 **d**	2.871 **ab**	35.196 **a**	9.911 **a**	4.045 **b**	0.217 **f**	2415.156 **a**
Blue lupin	Raw	93.816 **e**	2.706 **b**	29.329 **c**	4.062 **e**	6.987 **a**	1.011 **b**	1845.291 **d**
	Germinated	94.805 **b**	2.750 **ab**	32.342 **b**	4.993 **d**	6.251 **a**	1.205 **a**	2016.042 **c**
	Autoclaved	93.996 **e**	3.047 **a**	29.804 **c**	6.719 **c**	4.413 **b**	0.563 **e**	1965.850 **c**
Variety	White lupin	94.766 **A**	2.848	35.454 **A**	8.316 **A**	4.535 **B**	0.572 **B**	2322.568 **A**
	Blue lupin	94.206 **B**	2.834	30.492 **B**	5.258 **B**	5.884 **A**	0.927 **A**	1942.394 **B**
Process	Raw	94.200 **^b^**	2.810	32.249 **^b^**	5.529 **^c^**	5.884 **^a^**	0.832 **^b^**	2028.734 **^b^**
	Germinated	95.080 **^a^**	2.755	34.170 **^a^**	6.517 **^b^**	5.516 **^a^**	1.025 **^a^**	2178.207 ^a^
	Autoclaved	94.177 **^b^**	2.959	32.500 **^b^**	8.315 **^a^**	4.229 **^b^**	0.390 **^c^**	2190.503 ^a^
SEM		0.126	0.043	0.662	0.467	0.279	0.078	50.917
		*p* levels						
Variety (V)		0.000	0.860	0.000	0.000	0.000	0.000	0.000
Process (Prc)		0.000	0.130	0.001	0.000	0.001	0.000	0.000
V × Prc		0.021	0.178	0.051	0.680	0.053	0.771	0.157

**a**–**f**: Values in the same column with different letters are found to be statistically significant (*p* < 0.01). **A**,**B**: Values shown with different letters in the same column are statistically significant (*p* < 0.01). **^a^**^–**c**^: Values with different letters in the same column are statistically significant (*p* < 0.01). DM: dry matter; Ash: crude ash; CP: crude protein; EE: ether extract; ME: metabolizable energy; SEM: standard error of mean.

**Table 2:** Nutrient ingredients of white and blue lupin grains (part 2).

Main Effects	Treatments	CF (%DM)	NDF (g/kg DM)	ADF (g/kg DM)	ADL (g/kg DM)	Viscosity (cP = mPa.s)	Tot. Alkaloids (mg/g)
White lupin	Raw	8.316 **c**	158.482 **e**	138.092 **d**	8.547 **d**	43.847 **b**	50.796 **a**
	Germinated	11.185 **b**	220.673 **b**	127.389 **e**	11.921 **b**	43.000 **b**	14.893 **e**
	Autoclaved	8.467 **c**	171.274 **d**	123.382 **e**	8.204 **d**	43.540 **b**	36.260 **c**
Blue lupin	Raw	11.750 **b**	233.719 **a**	179.349 **b**	9.421 **c**	43.520 **b**	48.293 **b**
	Germinated	11.479 **b**	237.688 **a**	162.335 **c**	19.349 **a**	45.870 **a**	9.600 **f**
	Autoclaved	18.822 **a**	195.634 **c**	222.483 **a**	6.664 **e**	43.660 **b**	27.543 **d**
Variety	White lupin	9.323 **B**	183.476 **B**	129.621 **B**	9.557 **B**	43.462 **B**	33.983 **A**
	Blue lupin	14.017 **A**	222.347 **A**	188.056 **A**	11.811 **A**	44.350 **A**	28.479 **B**
Process	Raw	10.033 **^c^**	196.100 **^b^**	158.721 **^b^**	8.984 **^b^**	43.683	49.545 **^a^**
	Germinated	11.332 **^b^**	229.181 **^a^**	144.862 **^c^**	15.635 **^a^**	44.435	12.247 **^c^**
	Autoclaved	13.645 **^a^**	183.454 **^c^**	172.932 **^a^**	7.434 **^c^**	43.600	31.902 **^b^**
SEM		0.851	7.364	8.417	1.019	0.259	3.770
		*p* levels					
Variety (V)		0.000	0.000	0.000	0.000	0.016	0.000
Process (Prc)		0.000	0.000	0.000	0.0000	0.100	0.000
V × Prc		0.000	0.000	0.000	0.000	0.003	0.000

**a**–**f**: Values in the same column with different letters are found to be statistically significant (*p* < 0.01). **A**,**B**: Values shown with different letters in the same column are statistically significant (*p* < 0.01). **^a^**^–**c**^: Values with different letters in the same column are statistically significant (*p* < 0.01). CF: crude fiber; NDF: neutral detergent fiber; ADF: acid detergent fiber; ADL: acid detergent lignin; Tot. alkaloids: total alkaloids; SEM: standard error of the mean.

**Table 3:** Quinolizidine alkaloid levels of raw and processed white lupin (*Lupinus albus*) grains (#).

Quinolizidine Alkaloids (QA)	Raw Seeds (mg/g)	After Germination (mg/g)	After Autoclaving (mg/g)
Lupanine	>0.5	>0.5	>0.5
13α- Hydroxylupanine	>0.5	>0.5	>0.5
Cytisine	<0.01 *	<0.01 *	<0.01 *
Sparteine	0.013 ± 0.0052	<0.01 *	0.020 ± 0.008
Angustifoline	0.260 ± 0.1	0.180 ± 0.072	0.160 ± 0.064
Lupinine	<0.01 *	<0.01 *	<0.01 *
Multiflorine	>0.5	>0.5	>0.5
α-Isolupanine	0.170 ± 0.068	0.120 ± 0.048	0.120 ± 0.048
Sum of all positive QA	>1.943	>1.800	>1.800

* = Below indicated quantification level. (#) = Eurofins WEJ Contaminants GmbH (Hamburg) is accredited for this test. Result ± expanded measurement uncertainty (95%; k = 2); sampling not included. Color changes from light to dark in the table indicate the increase in alkaloid values.

**Table 4:** Quinolizidine alkaloid levels of raw and processed blue lupin (*Lupinus angustifolius*) grains (#).

Quinolizidine Alkaloids (QA)	Raw Seeds (mg/g)	After Germination (mg/g)	After Autoclaving (mg/g)
Lupanine	0.490	0.350 ± 0.140	0.390 ± 0.160
13α- Hydroxylupanine	0.260	0.190 ± 0.076	0.220 ± 0.088
Cytisine	<0.01 *	<0.01 *	<0.01 *
Sparteine	<0.01 *	<0.01 *	<0.01 *
Angustifoline	0.066	0.020 ± 0.0080	0.037 ± 0.015
Lupinine	<0.01 *	<0.01 *	<0.01 *
Multiflorine	<0.01 *	<0.01 *	<0.01 *
α-Isolupanine	0.078	0.049 ± 0.020	0.058 ± 0.023
Sum of all positive QA	0.894	0.609 ± 0.244	0.705 ± 0.282

* = Below indicated quantification level. (#) = Eurofins WEJ Contaminants GmbH (Hamburg) is accredited for this test. Result ± expanded measurement uncertainty (95%; k = 2); sampling not included. Color changes from light to dark in the table indicate the increase in alkaloid values.

**Table 5:** Antioxidant (ABTS and DPPH), phenolic compounds (PC), β-carotene, and color analysis results of white and blue lupin grains.

Main Effects	Treatments	ABTS (mg/mL)	DPPH (IC_50_ µL)	PC (mg GAE/L)	β-Carotene (mg/100 g)	Color		
						L*	a*	b*
White lupin	Raw	38.235 **b**	132.785 **cd**	1014.159 **c**	2.535 **d**	85.277 **a**	3.223 **b**	31.830 **c**
	Germinated	35.335 **c**	109.704 **d**	1187.809 **a**	4.709 **a**	84.950 **b**	1.920 **e**	30.933 **d**
	Autoclaved	42.697 **a**	79.386 **e**	1055.429 **b**	3.599 **c**	82.063 **c**	4.167 **a**	35.373 **a**
Blue lupin	Raw	22.713 **d**	150.066 **c**	256.857 **d**	2.316 **d**	81.750 **d**	2.150 **d**	34.280 **b**
	Germinated	18.691 **e**	296.534 **a**	188.286 **e**	4.088 **b**	81.690 **d**	0.453 **f**	31.033 **d**
	Autoclaved	18.245 **f**	236.025 **b**	209.714 **e**	2.414 **d**	80.063 **e**	2.357 **c**	31.930 **c**
Variety	White lupin	38.755 **A**	107.292 **B**	1085.799 **A**	3.614 **A**	84.097 **A**	3.103 **A**	32.712 **A**
	Blue lupin	19.883 **B**	227.542 **A**	218.286 **B**	2.939 **B**	81.168 **B**	1.653 **B**	32.414 **B**
Process	Raw	30.474 **^a^**	141.426 **^b^**	635.508 **^b^**	2.425 **^c^**	83.513 **^a^**	2.687 **^b^**	33.055 **^b^**
	Germinated	27.013 **^b^**	203.119 **^a^**	688.047 **^a^**	4.399 **^a^**	83.320 **^b^**	1.187 **^c^**	30.983 **^c^**
	Autoclaved	30.471 **^a^**	157.705 **^b^**	632.571 **^b^**	3.007 **^b^**	81.063 **^c^**	3.262 **^a^**	33.652 **^a^**
SEM		2.373	17.971	99.013	0.226	0.454	0.278	0.408
		*p* levels						
Variety (V)		0.000	0.000	0.000	0.000	0.000	0.000	0.014
Process (Prc)		0.000	0.0001	0.000	0.000	0.000	0.000	0.000
V × Prc		0.000	0.000	0.000	0.009	0.000	0.000	0.000

**a**–**f**: Values shown with different letters in the same column are statistically significant (*p* < 0.01). **A**,**B**: Values with different letters in the same column are statistically significant (*p* < 0.01). **^a^**^–**c**^: Values that are indicated by different letters in the same column are statistically significant (*p* < 0.01). PC: phenolic compounds, GAE: gallic acid equivalents, SEM: standard error of the mean.

**Table 6:** Fatty acid compositions (%) of white and blue lupin grains.

Fatty Acids (FAs) (%)	Short Name	White Lupin			Blue Lupin			Variety (V)		Process (Prc)				*p* Levels		
		Raw	Germinated	Autoclaved	Raw	Germinated	Autoclaved	White	Blue	Raw	Germinated	Autoclaved	SEM	V	Prc	V × Prc
Palmitic acid	C16:0	7.654 **b**	7.605 **b**	7.698 **b**	10.801 **a**	10.648 **a**	10.638 **a**	7.652 **B**	10.696 **A**	9.227	9.126	9.168	0.483	0.000	0.952	0.955
Stearic acid	C18:0	2.316 **b**	2.042 **b**	2.334 **b**	6.733 **a**	5.777 **a**	5.897 **a**	2.231 **B**	6.136 **A**	4.524	3.910	4.115	0.557	0.000	0.185	0.396
Oleic acid	C18:1n9c	53.875 **a**	51.331 **b**	53.814 **a**	38.882 **c**	38.701 **c**	39.444 **c**	53.007 **A**	39.009 **B**	46.378 **^ab^**	45.016 **^b^**	46.629 **^a^**	1.826	0.000	0.055	0.172
Linoleic acid	C18:2n6c	15.983 **d**	18.561 **c**	15.889 **d**	37.991 **a**	36.332 **b**	38.638 **a**	16.811 **B**	37.653 **A**	26.987	27.447	27.264	2.681	0.000	0.551	0.001
γ-linolenic acid	C18:3n6	12.513 **a**	12.651 **a**	12.505 **a**	2.805 **b**	3.626 **b**	2.937 **b**	12.557 **A**	3.122 **B**	7.659	8.139	7.721	1.291	0.000	0.698	0.837
Arachidic acid	C20:0	1.116 **ab**	1.084 **b**	1.143 **a**	0.755 **d**	0.817 **c**	0.790 **cd**	1.115 **A**	0.787 **B**	0.936 **^b^**	0.950 **^ab^**	0.967 **^a^**	0.045	0.000	0.088	0.006
Behenic acid	C22:0	3.793 **a**	3.855 **a**	3.835 **a**	1.620 **b**	1.711 **b**	1.656 **b**	3.828 **A**	1.662 **B**	2.706	2.783	2.746	0.287	0.000	0.501	0.960
Erucic acid	C22:1n9	1.821 **a**	1.952 **a**	1.898 **a**	0.000 **b**	0.000 **b**	0.000 **b**	1.890 **A**	0.000 **B**	0.910	0.976	0.949	0.253	0.000	0.526	0.526
DHA	C22:6n3	0.000 **b**	0.000 **b**	0.000 **b**	0.000 **b**	1.056 **a**	0.000 **b**	0.000 **B**	0.352 **A**	0.000 **^b^**	0.528 **^a^**	0.000 **^b^**	0.116	0.000	0.000	0.000
Lignoceric acid	C24:0	0.929 **a**	0.920 **a**	0.883 **a**	0.415 **b**	0.000 **c**	0.000 **c**	0.911 **A**	0.138 **B**	0.672 **^a^**	0.460 **^b^**	0.442 **^b^**	0.112	0.000	0.000	0.000
Nervonic acid	C24:1	0.000 **b**	0.000 **b**	0.000 **b**	0.000 **b**	1.768 **a**	0.000 **b**	0.000 **B**	0.589 **A**	0.000 **^b^**	0.884 **^a^**	0.000 **^b^**	0.196	0.002	0.000	0.000
Saturated FA (%)		15.808 b	15.505 **b**	15.892 **b**	20.323 **a**	18.952 **a**	18.982 **a**	15.735 **B**	19.419 **A**	18.066	17.229	17.437	0.596	0.000	0.330	0.451
Monounsaturated FA (%)		55.696 a	53.282 **b**	55.713 **a**	38.882 **c**	40.180 **c**	39.444 **c**	54.897 **A**	39.502 **B**	47.289	46.731	47.578	1.989	0.000	0.343	0.017
Polyunsaturated FA (%)		28.496 b	31.212 **b**	28.395 **b**	40.796 **a**	40.868 **a**	41.575 **a**	29.368 **B**	41.079 **A**	34.646	36.040	34.985	1.530	0.000	0.308	0.188

**a**–**d**: Values shown with different letters in the same row are statistically significant (*p* < 0.01). **A**,**B**: Different letters in the same row indicate statistically significant differences (*p* < 0.01). **^a^**^,**b**^: Values that are indicated by different letters in the same row are statistically significant (*p* < 0.01). SEM: standard error of the mean. V: lupin varieties. Prc: process, DHA: docosahexaenoic acid.

## Data Availability

The data that support the findings of this study are available from the corresponding author, upon reasonable request.
